# 855. Persistence on F/TAF versus F/TDF for HIV Pre-Exposure Prophylaxis: A Real-World Evidence Analysis in the United States

**DOI:** 10.1093/ofid/ofab466.1050

**Published:** 2021-12-04

**Authors:** Li Tao, Valentina Shvachko, Moupali Das, Christoph C Carter, Jared Baeten, David Magnuson

**Affiliations:** Gilead Science, Inc., Foster City, California

## Abstract

**Background:**

Persistence to preexposure prophylaxis (PrEP) is an important determinant of its efficacy, but evidence on real-world persistence is lacking. This study assesses adherence to F/TDF and F/TAF for PrEP both in terms of discontinuation and re-initiation patterns.

**Methods:**

We identified HIV-negative individuals in the United States who initiated F/TDF or F/TAF for PrEP between October 2019 and December 2020 from a de-identified prescription claims database; users taking generic F/TDF were excluded. Non-persistence was defined as a prescription fill gap of >30 days; discontinuation included switch from F/TDF to F/TAF or F/TAF to F/TDF. We used survival analyses to estimate persistence, Cox regressions to compare the hazard ratios (HR) of discontinuation, and logistic regression to compare the odds ratios of re-initiation after discontinuation.

**Results:**

Among F/TAF users (N=82,402) median age at PrEP initiation was 35 years (interquartile range [IQR] 28−47) and median PrEP persistence was 4 months (IQR 1.8-8.9), compared to 31 years (IQR 25−40) and 2 months (IQR 1.0-3.8) for F/TDF users (N=48,501). PrEP persistence at 60 and 90 days was higher among F/TAF users than F/TDF users (Figure). F/TDF users were 2.5 times more likely to discontinue than F/TAF users, with more marked differences in older users than that in younger users (*p* for interaction between discontinuation and age group < 0.01, Table). We also observed a higher rate of discontinuation of F/TDF versus F/TAF if PrEP was prescribed by internal medicine or infectious disease physicians than by family medicine physicians (data not shown). After discontinuation, F/TAF users were 1.7 times more likely than F/TDF users to re-initiate PrEP; the association was not different by age.

Persistence rates of F/TAF and F/TDF for PrEP by time of PrEP initiation

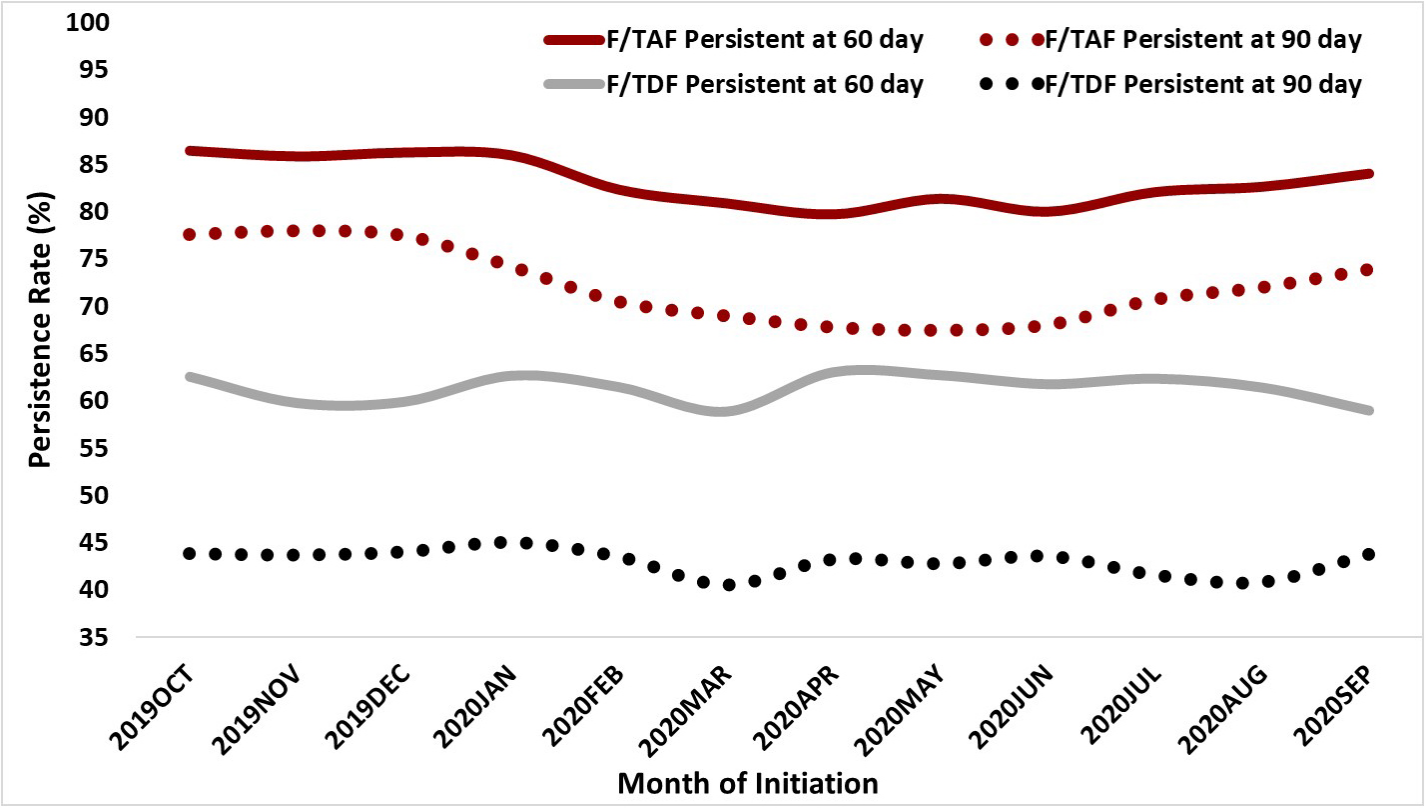

Hazard ratios (HR) and corresponding 95% confidence intervals (CI) of non-persistence and odds ratios (OR) of re-initiation after discontinuation for users of F/TAF and F/TDF for PrEP in the US, Oct 2019 – Dec 2020

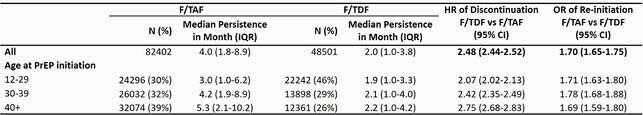

**Conclusion:**

In this real-world analysis, the F/TAF for PrEP regimen was associated with higher persistence and re-initiation than F/TDF for PrEP. These findings underscore the dynamic nature of PrEP utilization in the real-world and the importance of interventions aimed at improving PrEP persistence and re-initiation in people who would benefit from PrEP.

**Disclosures:**

**Li Tao, MD, PhD**, **Gilead Sciences Inc** (Employee, Shareholder) **Valentina Shvachko, MS**, **Gilead Sciences Inc** (Employee, Shareholder) **Moupali Das, MD**, **Gilead Sciences Inc.** (Employee, Shareholder) **Christoph C. Carter, MD**, **Gilead Sciences Inc.** (Employee, Shareholder) **Jared Baeten, MD, PHD**, **Gilead Sciences Inc.** (Employee, Shareholder) **David Magnuson, PharmD**, **Gilead Sciences Inc** (Employee, Shareholder)

